# Supplementary material for: The Effects of Fecal Microbial Transplantation on the Symptoms in Autism Spectrum Disorder, Gut Microbiota and Metabolites: A Scoping Review
Source: Microorganisms. 2025 May 31;13(6):1290. doi: 10.3390/microorganisms13061290 (PMC12195398; doi:10.3390/microorganisms13061290)
Supplement: Supplementary file 1 [file microorganisms-13-01290-s001.zip › microorganisms-3623251-supplementary.pdf]

Supplement Table S1. Characteristics of the clinical scales applied for assessment of clinical symptoms related to ASD.

Scale	Score Range (Min–Max)	Administered By	Purpose / Measures
**ABC (Autism Behavior Checklist)**	0–158	Parent / Caregiver	Measures problem behaviors in children with developmental disorders.
**ATEC (Autism Treatment Evaluation Checklist)**	0–179	Parent / Caregiver	Tracks treatment effectiveness for ASD—speech, sociability, behavior, health.
**CARS (Childhood Autism Rating Scale)**	15–60+	Clinician	Evaluates the severity of autism symptoms across 15 areas.
**CHAT-23**	0–23	Parent & Professional	Screens early autism signs through social/communication behavior.
**CNBS-R2016**	Varies by domain	Clinician	Chinese scale for assessing neuropsychological and behavioral development.
**HAMA (Hamilton Anxiety Rating Scale)**	0–56	Clinician	Measures the severity of anxiety symptoms.
**HAMD (Hamilton Depression Rating Scale)**	0–52	Clinician	Evaluates the severity of depressive symptoms.
**PGI-II (Patient Global Impression – Improvement)**	1–7	Clinician / Self-report	Assesses patient's perception of improvement after treatment.
**SAS (Self-Rating Anxiety Scale)**	20–80	Self-report	Screens for anxiety levels based on somatic and cognitive symptoms.
**SCL-90 (Symptom Checklist-90)**	0–450 (90 items, 0–5 each)	Self-report	Evaluates a broad range of psychological problems and symptoms.

Scale	Score Range (Min–Max)	Administered By	Purpose / Measures
**SDSC (Sleep Disturbance Scale for Children)**	26–130	Parent	Evaluates sleep disturbances in six domains in children.
**SRS (Social Responsiveness Scale)**	0–195	Parent / Teacher	Assesses severity of social deficits in ASD—awareness, cognition, communication, etc.
**VABS-II (Vineland Adaptive Behavior Scales, 2nd ed.)**	Varies by age/domain	Clinician / Parent Interview	Measures adaptive behaviors for daily functioning (social, motor, communication).
